# Renal function in children treated for central nervous system malignancies

**DOI:** 10.1007/s00381-016-3130-2

**Published:** 2016-06-21

**Authors:** Katarzyna Musiol, Grażyna Sobol-Milejska, Łukasz Nowotka, Karolina Torba, Maria Kniażewska, Halina Wos

**Affiliations:** 1 Department of Pediatric Oncology, Hematology and Chemotherapy, Upper Silesia Children’s Care Health Centre, Medical University of Silesia, 40-752 Katowice, 16 Medykow Str., Poland; 2 Department of Nephrology, Upper Silesia Children’s Care Health Centre, Medical University of Silesia, Katowice, Poland

**Keywords:** Brain tumours, Kidney injury, Cystatin C, Beta-2 microglobulin, Neutrophil gelatinase-associated lipocalin

## Abstract

**Aim:**

The aim of the study was to evaluate renal function and to assess the usefulness of the following nephrotoxicity markers: cystatin C (CYS C), beta-2 microglobulin (B2MG) and neutrophil gelatinase-associated lipocalin (NGAL) in 38 (18 girls, 20 boys) children previously treated for central nervous system malignancy.

**Material:**

Median age at evaluation was 13.7 years (range 2.1–22 years). The mean follow-up time after the completion of chemotherapy was 3.2 years (range 0.16–6.5 years).

**Results:**

Subclinical chronic kidney disease (estimated glomerular filtration rate: eGFR 90–60 ml/min/1.73 m^2^) was found in 22 patients (58 %), while renal insufficiency (eGFR 30–60 ml/min/1.73 m^2^) was found in six children (16 %). It has been demonstrated statistically significant negative correlation between the eGFR and cystatin C concentration (*p* < 0.0001) and eGFR and beta-2 microglobulin concentration (*p* < 0.02). Conversely, there was no correlation between eGFR and NGAL. Thirteen children (34 %) developed drug-induced tubulopathy: decreased tubular reabsorption of phosphate (TRP) and renal tubular threshold for phosphate (Tmp/GFR).

**Conclusion:**

Children treated for CNS tumours often develop drug-induced chronic renal disease, involving the glomeruli and/or renal tubules. Cystatin C and beta-2 microglobulin seemed to be good markers for chronic kidney damage in these patients, which is probably not true for NGAL.

## Introduction

Central nervous system (CNS) tumours are, after leukaemias, the most frequent malignancy in children. The number of children successfully treated for different malignancies has been increasing in recent years. Current 5-year survival rates of 73.3 % for childhood brain tumours suggest that the majority of children diagnosed with brain tumours will become long-term survivors [1]. Improved treatment outcomes can be attributed to the aggressive, multidrug chemotherapy, which is associated with drug-induced toxicity as one of the main adverse effects. Investigators from the Childhood Cancer Survivor Study (CCSS) reported that in 30 years after the cancer diagnosis, 73.4 % of survivors suffer from at least one chronic disease and they are eight times more likely to develop severe or life-threatening chronic health conditions as compared to their siblings [2]. The highest number of drug-induced complications is found in children treated for CNS tumours [2]. Specific late effects frequently observed in brain tumour survivors include neurotoxicity, neuro-cognitive deficits, endocrine disorders, obesity, secondary malignancy and kidney injury [1, 3–5].

Nephrotoxicity constitutes approximately 30 %, and according to some authors, up to 70 % of cancer treatment complications [6]. Platinum derivatives and alkylating agents, both known for their well-established nephrotoxicity, are primary chemotherapeutics used in the treatment of brain tumours [6, 7].

Nephrotoxicity of cisplatin and carboplatin is due to uptake of these drugs by the renal tubule cells. They cause damage to nuclear and mitochondrial DNA, activation of apoptosis and mediators of inflammation. These mechanisms result in kidney damage that can lead to acute kidney injury (AKI) and chronic lesions: fibrosis and tubular damage presenting with hypomagnesemia, hypocalcemia and distal renal tubular acidosis [7].

Alkylating agents: ifosfamide (IFA) and cyclophosfamide (CPA) act by alkylation of DNA. The side effect of IFA may be Fanconi syndrome—global proximal tubulopathy of the kidney that results in wasting of phosphate, calcium, uric acid and bicarbonates, as well as hyperaminoaciduria and glucosuria with a normal serum glucose level [8]. Adverse drug reaction from CPA is haemorrhagic cystitis.

Early detection and prompt treatment of chemotherapy-induced complications, including kidney injury, significantly affect the quality of life of cancer survivors.

Serum creatinine assay is the most common marker of kidney injury used in clinical practice. However, serum creatinine level fluctuations are neither sensitive nor specific response to slight changes of glomerular filtration rate (GFR). Hence, it is vital to search for the newer and better parameters, which correlate with the severity of kidney injury. In this regard, neutrophil gelatinase-associated lipocalin (NGAL), cystatin C (CYS C), beta-2 microglobulin (B2MG) and interleukin-18 (Il-18) appear now the most promising parameters [9, 10]. In many studies, these markers have been shown to be good indicators of AKI [11], but their role in the evaluation of chronic renal failure is still under investigation.

The aim of the study was to evaluate renal function in children previously treated for CNS malignancy and to assess the usefulness of the following chronic nephrotoxicity markers: CYS C, B2MG and NGAL in this patient population.

## Material and methods

### Study group

Data on 38 children (18 girls, 20 boys) previously treated for CNS tumour were analysed. The median age at diagnosis of CNS tumour was 9.75 years (range 0.92–17.7 years). Median age at evaluation was 13.7 years (range 2.1–22 years). The mean follow-up time after the completion of chemotherapy was 3.2 years (range 0.16–6.5 years). All patients were treated in the Department of Paediatric Oncology, Haematology and Chemotherapy at the Medical University of Silesia in Katowice between 2004 and 2013 and evaluated between January and June 2013 (Table 1).

**Table 1:** Clinical characteristics of the patients

Age at diagnosis (years)	9.75 (0.92–17.75)
Age at the time of investigation (years)	13.7 (2.1–22.0)
Sex	
Boys	20 (52 %)
Girls	18 (48 %)
Height (cm)	151.2 (95.5–190.5)
Height SDS	−0.95 (−4.5-1.8)
Weight (kg)	46.3 (15–79)
BMI SDS	0.06 (−2.9–0.87)
SBP (mmHg)	100.56 (88–135)
DBP (mm/Hg)	57.95 (43–87)
Histopathological examination:	
Medulloblastoma	19 (50 %)
Ependymoma anaplasticum	4 (10 %)
Astrocytoma anaplasticum	4 (10 %)
Oligodendroglioma anaplasticum	1 (3 %)
Oligoastrocytoma anaplasticum	2 (5 %)
Primitive neuroectodermaal tumour	1 (3 %)
Glioblastoma multiforme	2 (5 %)
Germinoma	4 (10 %)
Carcinoma plexus choroidei	1 (3 %)
Tumor location:	
Posterior fossa	19 (50 %)
Hemispheric location	12 (31 %)
Middle line	6 (15 %)
Brain stem	1 (3 %)
Resection:	
Total resection	12 (31 %)
Gross total resection	7 (18 %)
Partial resection	18 (47 %)
Biopsy	1 (3 %)
Radiotherapy spinal lumbar and sacral	Yes 22 (57 %)
	No 16 (42 %)

*SDS* standard deviation score, *SBP* systolic blood pressure, *DBP* diastolic blood pressure, *BMI* body mass index

The brain tumour treatment was in line with the Standardized and Modified Programme of Diagnosis and Comprehensive Treatment of Central Nervous System Tumours in Children (2007), recommended by the Polish Paediatric Group for Infant Neuro-oncology. It involved multidrug chemotherapy and radiotherapy administered in line with the histopathological diagnosis. Most children received both platinum compounds and alkylating agents (Table 2). Following protocols were applied in patients depending on their age and the type of neoplasm:

**Table 2:** Nephrotoxic chemotherapy used in the course of oncological treatment depending on the histopathological examination

Nephrotoxic chemotherapy	Pathomorphological evaluation	Number of patients
Ciplatin, carboplatin, IFA	MBL-HRG, PNET, EA,	14
Ciplatin, carboplatin, CPA	MBL-SRG	9
Ciplatin, CPA	Children under 3 years of age	4
Ciplatin, IFA	HGG	6
Ciplatin, carboplatin	Germ cell tumor	4
Carboplatin	Carcinoma plexus choroidei	1

*MBL-HRG* medulloblastoma high risk group, *PNET* primitive neuroectodermal tumor, *EA* ependymoma anaplasticum, *MBL-SRG* medulloblastoma slow risk group, *HGG* high-grade glioma

Protocol I: (vincristine, etoposide, carboplatin, cyclophosphamide, ifosfamide, lomustin and cisplatin)—in children with medulloblastoma, PNET and anaplastic ependymoma.
Protocol II: (etoposide, ifosfamide, adriamycin, cisplatin, vincristin, lomustin and temodal)—in children with high-grade glioma.
Protocol III: (cisplatin, etoposide, carboplatin and vincristine)—in children under 3 years of age.
Protocol V: (etoposide, vinblastine, bleomycin, cisplatin and carboplatin)—in children with germ cell tumour.

Majority of children according the Therapeutic Protocol have received cisplatin as 1-h bolus infusions on five consecutive days (5 × 20 mg/m^2^) during initial therapy (before radiotherapy) and as 1-h bolus infusion on 1 day (1 × 75 mg/m^2^) in the course of maintenance therapy (after radiotherapy). No one received long-term ciplatin infusions. Appropriate hydration during chemotherapy was conducted to reduce the incidence of cisplatin-induced nephrotoxicity. Mannitol and not furosemide was administered during hydration to decrease cisplatin-induced nephrotoxicity.

None of the patients received intravenous radiographic contrast; in all patients, gadolinium-based contrast agents were applied during magnetic resonance imaging. One patient during febrile neutropenia has received aminoglycoside (during 7 days) because of respiratory tract infection, and one child received sulfonamides because of Pneumocytis carini infection.

All patients had normal renal function before chemotherapy measured by a new Schwartz formula (mean eGFR 101 ml/min/1.73 m^2^).

### Laboratory methods and analysis

All enrolled patients underwent diagnostic evaluation including blood pressure measurement, blood sample collection for serum creatinine and electrolyte level (serum magnesium, phosphorus, calcium, sodium, potassium, glucose) and CYS C, NGAL and B2MG assays. The 24-h urine collection was performed in order to assess the loss of phosphorus, sodium, magnesium and calcium ions (in mg/kg/24 h). Furthermore, plasma and urine osmolality, the urine specific gravity, pH, presence of glucose, protein and albumin were determined. Microalbuminuria was defined as losing more than 15 μg of albumines per minute during 24-h urine collection. All the tests were performed according to standardized routine methods in a hospital laboratory with the Olympus 800u apparatus. Additionally, an abdominal ultrasound scan was performed in each patient.

Glomerular function was evaluated by determining serum creatinine and by estimating GFR with Schwartz formula and updated CKD Schwartz Equation (new Schwartz formula) [12].

The Schwartz formula was defined as eGFR in ml/min/1.73 m^2^ = height of child [cm] × k/serum creatinine [mg/dl]; *k*- = 0.413.

The new Schwartz formula equation was defined as 39.2 × (height [m]/serum creatinine [mg/dl]) 0.516 × (1.8/CYS C serum concentration [mg/L] 0.294 × (30/BUN [mg/dl]) 0.169 × (1,099 males/1 female) × (height of child [m]/1.4) 0.1888 [12, 13].

Nephrotoxicity was defined according to indicates of National Kidney Foundation (http://www.kidney.org/professionals/KDOQI/guideline-/ckd):

- Mild decrease of eGFR (eGFR 90–60 ml/min/1.73 m^2^)-stage 2 of chronic kidney damage (CKD)
- Moderate decrease eGFR (eGFR 60–30 ml/min/1.73 m^2^)-stage 3 of CKD
- Severe reduction eGFR (eGFR 30–15 ml/min/1.73 m^2^)-stage 4 of CKD
- Kidney failure (eGFR < 15 ml/min/1.73 m^2^)-stage 5 of CKD

Tubular function was estimated by calculating the ratio of the tubular reabsorption of phosphate (TRP) and renal tubular threshold for phosphate (Tmp/GFR). TRP was defined as:

- TRP (%) = 1 − (urine phosphorus concentration/urine creatinine concentration) × (serum creatinine concentration/serum phosphorus concentration) × 100. TRP dysfunction was defined as TRP < 85 %.

Tmp/GFR—a quotient of maximal rate of tubular phosphate reabsorption and the glomerular filtration rate. References ranges for children aged 2–15 years were 1.15–2.6 mmol/l (21–47 mg/dl) according to http://www.baspath.co.uk/test_directory/tindex/TmPGFR.htm [14].

Simultaneously, the serum levels of CYS C, B2MG and NGAL were measured.

The level of B2MG was assessed with the immunoenzymatic method and using commercial sets of B2-Mikroglobulin Elisa Kit by Immundiagnostik AG, Germany. The detection limit was defined as Bo + 2 SD and set to 0.1 mg/l.

CYS C was assayed by a commercial kit: Human Cystatin C ELISA by BioVendor, Czech. The limit of detection is calculated from the real CYS C values in wells and is 0.25 ng/ml.

The level of NGAL was assessed with the immunoenzymatic method and using commercial sets of Human lipocalin-2/ngal ELISA by BioVendor, Czech. The limit of detection is calculated from the real NGAL values in wells and is 0.02 ng/ml.

The results of nephrotoxic markers were correlated with clinical data: age at diagnosis, a type and total doses of chemotherapy and time lapse from treatment completion.

The study was approved by the Ethical Committee of Medical University of Silesia in Katowice. The written informed consent was obtained from the parents of the participants.

### Statistical analysis

Most variables showed normal distribution (*p* > 0.05 in KS test) and were characterized as mean values (min–max value). Hypotheses were verified using the parametric Student *t* tests for independent and dependent variables, respectively. The continuous data was analysed using Mann–Whitney *U* or Kruskall–Wallis test. The Spearman correlation coefficient was used for correlation estimates. *P* value below 0.05 was considered statistically significant. All statistical analyses were performed using the STATISTICA data analysis software system version 11 (StatSoft, Inc. 2014 www.statsoft.com).

## Results

The mean GFR estimated using Schwartz formula in the whole group was 76 ml/min/1.73 m^2^, while GFR estimated using revised Schwartz formula was 63 ml/min/1.73 m^2^. There was a statistically significant difference between GFR determined using the Schwartz formula and the revised Schwartz formula equations (*p* < 0.0001).

Subclinical stage 2 of CKD (eGFR 90–60 ml/min/1.73 m^2^) was found in 22 patients (58 %), and stage 3 of CKD (eGFR 30–60 ml/min/1.73 m^2^) was found in six children (16 %). The results of investigations are shown in Table 3.

**Table 3:** Distribution of patients considering the degree of kidney damage

eGFR	
eGFR >90 ml/min/1.73 m^2^	eGFR 60–90 ml/min/1.73 m^2^
No nephrotoxicity	Stage 2 of nephrotoxicity
10 pts (26.5 %)	22 pts (58 %)

*eGFR* estimated glomerular filtration rate

No significant correlation between patient age at diagnosis, and the severity of kidney injury was observed. Similarly, no correlation between the GFR and time lapse from treatment completion was found.

The cumulative dose of nephrotoxic agents (IFA, CPA, cisplatin and carboplatin) did not correlate with the severity of kidney injury (Table 4).

**Table 4:** Correlation between eGFR and the cumulative dose of nephrotoxic agents

Drug cumulative dose	Cisplatin		Carboplatin		Ifosfamide		Cyclophosfamide
	<450 mg/m^2^	>450 mg/m^2^	<1100 mg/m^2^	>1100 mg/m^2^	0 g/m^2^	9–36 g/m^2^	0 g/m^2^
Number of patients	18	20	16	22	20	18	25
eGFR Shwartz	66	60	57	65	66	59	60
*p*	0.15	0.14	0.08	0.09	–	0.11	–

*eGFR* estimated glomerular filtration rate

There was no statistically significant difference in GFR between the subgroup of children treated with radiotherapy e.g. to the lumbar spine and those who did not receive radiotherapy.

Children with CKD (GFR < 60 ml/min/1.73 m^2^) showed significantly higher levels of CYS C (1500.21 vs 992.64 mg/L) and B2MG (2.06 vs 1.23 mg/L), as compared to the remaining subjects. NGAL levels were comparable in both subgroups (18.91 vs. 18.94 ng/ml) (Table 5).

**Table 5:** Serum Cys C, NGAL and B2MG among patients with and without nephrotoxicity

	Serum CysC (ng/ml)	Serum NGAL (ng/ml)	Serum B2MG (mg/l)
eGFR new Shwartz >60 ml/min/1.73 m^2^	992.64 (554.55–1642.9)	18.91 (8–44.7)	1.235 (0.75–2.45)
eGFR new Shwartz 30–60 ml/min/1.73 m2	1500.21 (728.36–2906.1)	18.94 (9.2–51.7)	2.068 (1.25–5.93)
*p*	<0.001	0.6	<0.01

*Cys C* cystatin C, *NGAL* neutrophil gelatinase-associated lipocalin, *B2MG* beta-2 microglobulin, *eGFR* estimated glomerular filtration rate

**Fig. 1:**
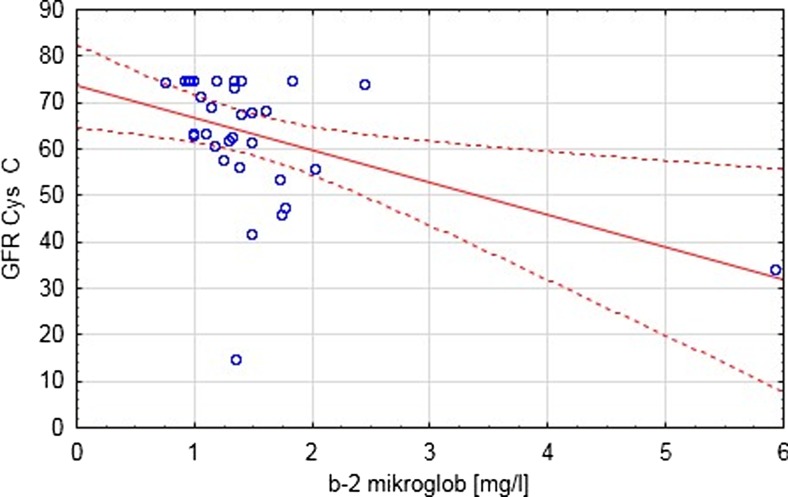
Correlation between eGFR C and B2 microglobulin (*p* < 0.02)

**Fig. 2:**
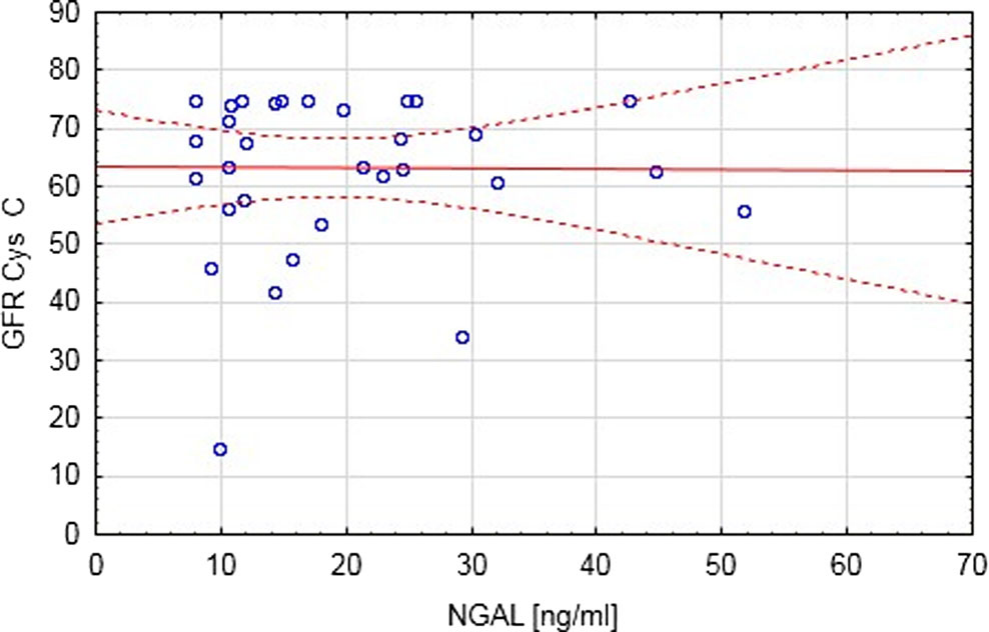
Correlation between eGFR C and NGAL (*p* = 0.6)

A statistically significant negative correlation between the eGFR estimated by Schwartz formula and CYS C levels (*p* < 0.003) was shown across the entire study group. The correlation between the eGFR estimated by the new Schwartz formula and CYS C (*p* < 0.0001) results from the equation itself. Furthermore, a correlation between the eGFR estimated using the new Schwartz formula and B2MG was shown (*p* < 0.02) (Fig. 1). Conversely, there was no correlation between eGFR and NGAL (*p* = 0.6) (Fig. 2).

Additionally, a strong positive correlation between serum CYS C level and B2MG (*p* < 0.001) was observed. However, no associations between CYS C and NGAL (*p* = 0.8) or B2MG and NGALwere found (*p* = 0.4).

Thirteen children (34 %) developed drug-induced tubulopathy: decreased tubular reabsorption of phosphate (TRP and TmP/GFR). It manifested as hypophosphatemia and hyperphosphaturia. Hypophosphatemia and hypomagnesaemia were found in 36 and 13 % of study subjects, respectively. Serum levels of calcium, sodium and potassium in all subjects were within the normal range. All urine samples tested negative for the presence of glucose and protein. Mild metabolic acidosis manifesting as slightly decreased blood pH and BE (acid–base equilibrium) was shown in 3 (1 %) patients (Table 6). Microalbuminuria was present in five children.

**Table 6:** Concentration of Mg, P, Ca and TRP, TmP/GFR in patients

	Patients	
	With tubulopathy	Without tubulopathy
Serum P (mmol/l)	1.17 (min: 0.96–max: 1.18)	1.28 (min: 1.0–max: 1.49)
Serum Mg (mmol/l)	0.86 (min: 0.54–max: 0.97)	0.79 (min: 0.58–max: 1.1)
Serum Ca (mmol/l)	2.55 (min:2.53–max: 2.67)	2.52 (min: 2.34–max: 2.64)
TRP (%)	Median:76.2 (min: 64–max: 84.9)	Median: 89.2 (min:85.1–max: 97)
TmP/GFR	Median: 0.83 (min: 0.55–max: 0.98)	Median: 1.09 (min: 0.88–max: 1.67)

*TRP* renal reabsorption of phosphate, *TmP/GFR* renal tubular threshold for phosphate, *GFR* glomerular filtration rate

CYS C and B2MG levels also showed a strong positive correlation with TRP and TmP/GFR (*p* < 0.001). There was no such correlation between NGAL and TRP (*p* = 0.7) or NGAL and TmP/GFR (*p* = 0.762).

Ultrasound image of kidneys appeared normal in all children. Except for one 22-year-old patient with average blood pressure of 137/87 mm/Hg, the blood pressure measurements were consistently normal across the entire group.

## Discussion

Currently, as the 5-year survival in children with different malignancies has reached almost 70 %, oncologists aim at offering cancer survivors the best possible quality of life. According to published data, 40 % of cancer survivors develop chronic conditions [15]. Brain tumour survivors are the most severely affected with late effects of cancer treatment [16]. The most common sequelae include endocrine dysfunction, cardiotoxicity, neurotoxicity and nephrotoxicity. According to some authors, chronic nephrotoxicity affects 30–70 % of children after previous antineoplastic therapy [6]. Since CKD leads to end-stage renal failure in many cases, its early diagnosis and appropriate treatment may have a significant impact on its course. However, potential preventive and therapeutic interventions are often delayed. Hence, prompt diagnosis during the prodromal stage is necessary, followed by the long-term observation.

Commonly used nephrotoxicity markers such as serum creatinine level have many limitations. Serum creatinine level depends on numerous confounding factors: muscle mass, sex, age, diet, muscle metabolism, body hydration and applied medications. Serum creatinine level is elevated as a result of damage to at least 50 % of kidney parenchyma. Moreover, elevated creatinine level is not specific for tubular damage [11].

### GFR

The eGFR is an estimator of functional nephron count used in clinical practice. It constitutes a basis for CKD staging [17]. Fitness level conditions endogenous creatinine and estimation of GFR. Furthermore, the determination of GFR using inulin (the gold standard for determining GFR) is associated with many obstacles, such as the need for repeated blood sampling, which may be troublesome particularly in paediatric population. Taking into consideration these reservations, the research for better parameters of GFR is ongoing. Mathematical equations involving additional parameters may increase diagnostic sensitivity of GFR in an individual patient. The National Kidney Foundation (NKF) recommends the Schwartz formula for estimating GFR in children (https://www.kidney.org/content/creatinine-based-%E2%80%9Cbedside-schwartz%E2%80%9D-equation-2009). In 2009, Schwartz published a new, revised equation to estimate GFR in children with CKD. The new formula is based on the serum concentration of creatinine, CYS C and uric nitrogen [12]. According to some authors, the simultaneous use of at least two methods (GFR estimated based on serum creatinine and CYS C levels) offers more reliable results [18].

As a part of our study, renal function in each patient was estimated using both Schwartz formula and revised Schwartz formula. The difference between the mean GFR estimated by Schwartz formula (76 ml/min/1.73 m^2^) and GFR estimated revised Schwartz formula (63 ml/min/1.73 m^2^) was statistically significant (*p* < 0.0001).

In this study, the laboratory signs of subclinical nephrotoxicity (eGFR of 90–60 ml/min/1.73 m^2^) were observed in 57.8 % of patients, which corresponds to the findings by other authors [19]. In our patient cohort, 15.7 % of cancer survivors had eGFR of 60–30 ml/min/1.73 m^2^ (including one individual with eGFR of 34 ml/min/1.73 m^2^). Skinner et al. found eGFR <60 ml/min/1.72 m^2^ in 13 % of their 25-person study group with malignant sarcoma treated with IFA before the end of 10-year follow-up [20]. Similarly, in a group of 63 patients treated with cisplatin, 11 % of subjects had decreased GFR at the end of the 10-year follow-up [7]. Oberlin et al. found decreased GFR <60 ml/min/1.73 m^2^ in 21 % of patients with GFR being as low as 50.1 ml/min/1.73 m^2^ in a single case. Their study group included children treated with IFA for soft tissue sarcomas and bone sarcomas [6]. In the study by Zubowska et al., 20 % of childhood cancer survivors developed CKD (GFR < 60 ml/min/1.72 m^2^) [19]. Most studies were carried out on mixed case groups including only a few brain tumour survivors [19, 20]. We have not found any studies focusing on late nephrotoxicity in patient samples consisting only of brain tumour survivors.

We did not find an association between the age at diagnosis and GFR in contrast to other authors who report correlations between CKD and patient age. Skinner [21] showed more severe proximal tubular toxicity in younger children treated with IFA, as compared to older children. However, the same author later found older age at treatment to be a risk factor for cisplatin nephrotoxicity [7]. There are also other papers emphasizing that patients younger than 5 years are more likely to develop CKD [21–23]. Perhaps the lack of correlation of GFR with age in our work results from the fact that an average age of children was 9.5 years (consistent with epidemiological data regarding the age of the incidence of malignant CNS tumours), and there were only two children under 5 years of age in the studied group.

The fact that our study failed to show relationship between specific factors such as age and total dose of administered drugs could also be related to the modest sample size.

We did not observe an association between the time interval from treatment completion and GFR. Similarly, Zubowska et al. did not observe the association between the follow-up time and nephropathy in a group observed for the mean period of 4.6 years [19]. Furthermore, other authors who carried out their studies at 10 years following chemotherapy completion did not observe kidney function deterioration with time [20]. However, Oberlin in her study on 138 patients who had completed chemotherapy at least 5 years earlier found a longer follow-up period to be an independent risk factor for abnormal GFR [6]. On the other hand, there are papers reporting spontaneous recovery of renal function after chemotherapy [25, 26].

Many authors indicate an association between kidney damage and cumulative dose of chemotherapeutics. It is true for both such platinum derivatives as carboplatin, cisplatin [25, 26] and IFA [6, 24].

We did not confirm an association between the total dose of chemotherapeutics (cisplatin, carboplatin, IFA and CPA) and GFR, which might potentially be attributed to the fact that all children in our study group received chemotherapy in line with approved treatment protocols involving simultaneous use of IFA and/or CPA as well as cisplatin and/or carboplatin. Multidrug chemotherapy may constitute a confounding factor. All quoted papers assessed nephrotoxicity after monotherapy. Other authors reported a significant effect of IFA on the kidney function when a cumulative dose of the drug exceeded 60 g/m^2^. Children in our study group did not receive such a high total dose of IFA. In the literature, there is no conclusive data on the toxic dose of cisplatin. Skinner showed toxic effect of a daily dose of cisplatin exceeding 40 mg/m^2^. There is the lack of data on toxic total doses of carboplatin.

There was no statistically significant difference in GFR between the subgroup of children treated with craniospinal radiation therapy and those who did not receive radiotherapy. In Zubowska paper, the higher frequency of nephrotoxicity was observed among the children after abdominal radiotherapy [19].

### Markers of kidney injury

There are many studies assessing new markers of early kidney damage, immediately after activation of a damaging factor [10]. Very few biomarkers exist for monitoring CKD.

Neutrophil gelatinase-associated lipocalin (NGAL) is a protein secreted to urine by the cells of the thick ascending limb of loop of Henle and a connecting tubule [27]. Based on the available published data NGAL is thought to be a novel, sensitive and specific early marker of acute kidney injury (AKI) [11]: in ischemic acute kidney injury [28, 29], in a septic shock [30], in contrast-induced nephropathy [31]. However, the role of measurement of NGAL in CKD is still unclear. Several recent studies have showed increased serum NGAL levels in cases with CKD [32–34]. Mitsnefes et al. showed that serum NGAL significantly correlated with cystatin C and both NGAL and cystatin C significantly correlated with eGFR in children with CKD stages 2–4 [35]. In our study, no significant association between plasma NGAL levels and eGFR was found, and NGAL levels in children with kidney injury were comparable to those in children with normal eGFR. In line with Forest Fire Theory [35], we consider NGAL to be a marker of an early as well as AKI. An initial elevation of NGAL level directly after the exposure to an insulting agent (“burning trees”) is followed by its decrease to the values comparable with those of healthy individuals (“burnt out trees”). Our findings correspond to those of Nikolas in a sample of 635 patients: NGAL levels in patients with CKD and those with normal kidney function were comparable [36]. Plasma NGAL measurements may be influenced by a number of coexisting variables such as chronic hypertension [37], systemic infection [38] and neoplasms [39]. Recent studies have showed that in the primary brain neoplasms, NGAL is over-expressed in tumours, which correlates with elevated serum and urine NGAL [40, 41]. Thus, it seems that the concentration of serum NGAL in children with cancer can be caused by many factors and not only resulting from kidney damage.

CYS C undergoes glomerular filtration and a complete reabsorption in proximal tubules. However, it is not involved in tubular secretion. The stability of the molecule of CYS C and the fact that its concentration in blood depends only on the glomerular filtration rate affects its high diagnostic efficiency [42]. CYS C has been studied intensively as a marker of kidney function in adults and in children. A number of cross-sectional studies have been published showing that serum CYS C concentration is more sensitive and correlate better to GFR than creatinine [43, 44]. Many reports emphasize that serum cystatin C is a better indicator of GFR than serum creatinine concentration both in patients with chronic kidney damage [45] including chemotherapy [46]) and in patients with acute kidney injury: in papers by Liang [47] and Krawczewski [48]. Cys C level increased as early as 12 h following the exposure to a nephrotoxic agent and preceded the elevation of creatinine level by 1–2 days. Similarly, we showed a statistically significant negative correlation between the eGFR estimated by Schwartz formula and CYS C levels (*p* < 0.003) across the entire study group. Children with CKD (GFR < 60 ml/min/1.73 m^2^) had significantly higher levels of CYS C as compared to the remaining subjects (*p* = 0.001).

Beta-2 microglobulin (B2MG) is a subunit of the major histocompatibility class I molecule produced by all nucleated cells [49]. Its small size (11.8 kDa) allows beta-2-M to pass through the glomerular membrane, but it is almost completely reabsorbed in the proximal tubules. Serum beta-2-M levels are elevated in diseases associated with the increased cell turnover. This assay offers improved diagnostic sensitivity for the detection of altered GFR as compared to serum creatinine [50]. B2MG level is also elevated in several benign conditions such as chronic inflammation, liver disease, some acute viral infections and a number of malignancies, especially haematologic malignancies associated with the B cell lineage [51]. Many studies showed a very strong association between plasma B2MG levels and GFR [52]. However, there are few reports evaluating the B2MG in children after chemotherapy. In our study, we observed a statistically significant negative correlation between GFR and B2MG levels (*p* < 0.02). Additionally, we observed a strong positive correlation between serum CYS C level and B2MG (*p* < 0.001). However, we did not observe an association between CYS C and NGAL (*p* = 0.8) or B2MG and NGAL (*p* = 0.4).

### Tubulopathy

Fanconi syndrome is characterized by a global transport defect in the proximal tubules of the kidney. The spectrum of tubular dysfunction varies in different patients, ranging from a generalized proximal tubulopathy to partial reabsorption defects in phosphorus, calcium, glucose, amino acids and bicarbonate. The mechanism by which IFA induces Fanconi syndrome has not been identified [8]. Distant effects of IFA tubulopathy in children may be growth retardation [24] and the bone disease rickets [53]. CPA, an isomer of IFA, displays only side effects in the form of haemorrhagic cystitis but not other nephrotoxicities.

In our study, 36 % of children presented with hypophosphatemia, which corresponds with the remaining signs of tubulopathy. Decreased fractional TRP (TRP < 85 %) and the decreased ratio of tubular maximum reabsorption of phosphate to GFR (TmP/GFR < 1.15) were found in 34 % of patients. The patients with hypophosphatemia also showed decreased tubular reabsorption and TmP/GFR. All subjects had normal serum and urine sodium, potassium and calcium levels. Among all patients, the osmolality of urine and plasma was normal. For this reason, we did not measure tubular handling of sodium, calcium and potassium. All urine samples tested were negative for the presence of glucose and protein. Three patients were found to have mild metabolic acidosis, which did not require intervention. Five children had microalbuminuria (including one patient with other symptoms of tubulopathy). In summary, 1/3 of children presented partial Fanconi syndrome, which clinically manifested itself mainly with hypophosphatemia and hyperphosphaturia. These findings confirm results obtained by other authors, who observed persistent dysfunction of renal proximal tubule cells in 5–10 % of children treated only with IFA [6, 23] and in 30–40 % of children treated with IFA and concurrent administration of cis or carboplatinum [24]. Patients included in these studies were mainly children after treatment of bone and soft tissue sarcomas, neuroblastoma and Wilms’ tumour. We have not found any studies focusing on tubular function assessment in patients after brain tumour therapy in children.

None of our patients experienced hemorrhagic cystitis caused by the CPA, which was probably associated with prophylactic administration of the uroprotectant mesna and intensive hydration during and after chemotherapy.

## Conlusions

Summing up, children treated for CNS tumours often develop drug-induced chronic renal disease, involving the glomeruli and/or renal tubules. The strength of our work is the fact that it was carried out on a homogeneous research group. The downside is a small size of the group, due to the fact it was conducted in a single centre and low survival rate of patients with the disease (the highest mortality rate of all cancers in children). Further study should be performed in larger group of patients, in multicenter studies. Longer time of follow-up is needed to constitute conclusions assessing the changes in the kidney in the course of time. Our data suggests the confirmation of the hypothesis that CYS C and B2MG can be used as markers of chronic kidney injury after chemotherapy in children, which is probably not true for NGAL. It also seems that none of these markers meets the criterion of universal indicator of kidney damage and it is necessary to develop a diagnostic panel to detect kidney injury in a preclinical phase.
